# Imatinib inhibits oral squamous cell carcinoma by suppressing the PI3K/AKT/mTOR signaling pathway

**DOI:** 10.7150/jca.88555

**Published:** 2024-01-01

**Authors:** Lei Ma, Ke Huang, Haibo Zhang, Eungyung Kim, Hyeonjin Kim, Zhibin Liu, Chae Yeon Kim, Kanghyun Park, Muhammad Atif Raza, Kirim Kim, Junkoo Yi, Yonghun Sung, Zae Young Ryoo, Yong-Gun Kim, Myoung Ok Kim

**Affiliations:** 1 Department of Animal Science and Biotechnology, Research Center for Horse Industry, Kyungpook National University, Sangju, Gyeongsangbuk-do 37224, Republic of Korea.; 2 College of Pharmacy, Henan University of Chinese Medicine, Zhengzhou, 450046, China.; 3 Department of Dental Hygiene, Kyungpook National University, Sangju, Republic of Korea.; 4 School of Animal Life Convergence Science, Hankyong National University, Anseong, 17579, Republic of Korea.; 5 Preclinical Research Center, Daegu-Gyeongbuk Medical Innovation Foundation (DGMIF), Daegu 41061, Republic of Korea.; 6 School of Life Sciences, BK21 FOUR KNU Creative BioResearch Group, Kyungpook National University, Daegu 41566, Republic of Korea; 7 Department of Periodontology, Kyungpook National University School of Dentistry, Daegu, South Korea.

**Keywords:** imatinib, oral squamous cell carcinoma, proliferation, viability, PI3K/AKT/mTOR signaling pathway

## Abstract

Oral squamous cell carcinoma (OSCC) is a prevalent oral and maxillofacial cancer with high mortality as OSCC cells readily invade tissues and metastasize to cervical lymph nodes. Although imatinib exhibits potential anticancer and remarkable clinical activities that therapeutically affect several cancer types, its specific impact on OSCC has yet to be fully explored. Therefore, this study investigated the potential anticancer effect of imatinib on OSCC cells and the underlying mechanisms. The Cell Counting Kit-8 was used to determine the impact of imatinib on cell viability. Then, morphological cell proliferation analysis was conducted to examine how imatinib impacted OSCC cell growth. Moreover, OSCC cell migration was determined through wound-healing assays, and colony formation abilities were investigated through the soft agar assay. Lastly, the effect of imatinib on OSCC cell apoptosis was verified with flow cytometry, and its inhibitory mechanism was confirmed through Western blot. Our results demonstrate that imatinib effectively inhibited OSCC cell proliferation and significantly curtailed OSCC cell viability in a time- and concentration-dependent manner. Furthermore, imatinib suppressed migration and colony formation while promoting OSCC cell apoptosis by enhancing p53, Bax, and PARP expression levels and reducing Bcl-2 expression. Imatinib also inhibited the PI3K/AKT/mTOR signaling pathway and induced OSCC cell apoptosis, demonstrating the potential of imatinib as a treatment for oral cancer.

## Introduction

Oral cancer (OC), predominantly in the head and neck, is characterized by malignant tissue development in the oral cavity 1. Approximately 500,000 new OC cases are diagnosed annually, with 75% localized in developing countries 2, and oral squamous cell carcinoma (OSCC) accounts for 90% of all OC cases 3. Chronic stimuli that induce OSCC include dental caries, excessive mouthwash usage, chewing tobacco, or betel quid. Early diagnosis through health screenings is crucial for preventing this fatal illness because early, treatable lesions are rarely symptomatic 4. As such, morbidity and mortality have surged worldwide due to missed opportunities for early diagnosis and treatment 5. Radiation, chemotherapy, and other therapies are currently utilized with surgery to treat patients 6. However, although considerable progress has been made, advanced OSCC individuals still receive a poor prognosis 7. Thus, in-depth research on molecular pathogenesis of OSCC is vital for identifying novel and effective biomarkers.

Imatinib selectively inhibits several tyrosine kinases, including Bcr-Abl, c-Kit, and the platelet-derived growth factor receptor, to block downstream signaling pathways critical for cancer cell survival and proliferation 8. In addition, imatinib induces cell cycle arrest and apoptosis in cancer cells. Studies have demonstrated that imatinib mediates endoplasmic reticulum stress-induced apoptosis in gastric cancer cells 9. Recent studies have also proposed that imatinib attenuates MMP-2 and MMP-14 expression levels in the P16 cell line, slowing SCC tumor growth and inducing apoptosis 10. Furthermore, imatinib and carboplatin combined inhibits vascular endothelial growth factor, platelet-derived growth factor (PDGF), and PDGF-R/ß expressions in HNSCC 11.

Therefore, considering its anticancer potential against various malignancies, this study explored the potential anticancer effect of imatinib on OSCC cells and the underlying mechanisms.

## Materials and Methods

### Ethical statement

The Kyungpook National University (KNU) Institutional Review Board (KNUDH-2022-07-02-00) approved the protocol of this study.

### Reagents and antibodies

First, various working concentrations of imatinib (>98% purity; Harvey Biotech Co., Beijing, China) were dissolved in dimethyl sulfoxide (DMSO), and the following primary antibodies were obtained from Santa Cruz Biotechnology Inc. (TX, USA): E-cadherin (67A4), N-cadherin (13A9), and β-actin (C4). In addition, PARP (#9532), Bax (#2772), P53 (#2524), phospho-AKT (#9271), AKT (#9272), phospho-PI3K (#17366), PI3K (#4249), Phospho-mTOR (#5536), mTOR (#2983), and Bcl-2 (#15071) were purchased from Cell Signaling Technology (MA, USA).

### Cell culture

Primary human gingival fibroblasts (HGnF) were obtained from three systemically and periodontally healthy individuals below 35 years of age using a previously described tissue explant technique 12. Primary cells derived from 1mm tissue samples were cultured in Dulbecco's Modified Eagle Medium (DMEM; Gibco™, NY, USA) supplemented with 10% fetal bovine serum (FBS; GenDEPOT, TX, USA), and 1% penicillin/streptomycin (PS; Gibco™, NY, USA). Cells that had not received more than seven passages were starved in a 0.3% FBS-containing medium before culturing under experimental conditions. Human OSCC cell lines (YD-10B and Ca9-22) were obtained from the Department of Oral Biology, College of Dentistry, Yonsei University, Seoul, Korea. OSCC cells were cultured in DMEM supplemented with 10% FBS and 1% PS and incubated in a humidified 5% CO_2_ atmosphere at 37 °C.

### Cell viability assay

Cells were seeded at 2 × 10^3^ cells per well in 96-well plates and cultured overnight in a complete medium. The attached cells were treated with different imatinib concentrations (0, 2.5, 5, 10, 20, and 40μM) in serum-free media over specific durations (0, 24, 48, 72, and 96h). Next, cell viability was assessed by adding 10μl of the Cell Counting Kit-8 (CCK-8; Dojindo, Japan) solution to each well, followed by incubation for 3 h at 37 °C. Absorbance was then measured at 570 nm using a microplate reader (Thermo Fisher Scientific, Waltham, MA, USA).

### Colony formation assay

A modified soft agar assay was used to investigate imatinib's effect on colony formation. Initially, growth media supplemented with different imatinib concentrations were prepared, and a bottom layer of 0.6% agar mixed with the respective imatinib concentration was plated in 6-well culture plates. Then, each well was supplemented with a complete growth medium top layer containing 8 × 10^3^ cells per well, corresponding imatinib concentrations (0, 2.5, 5, 10, 20, and 40μM), and 0.3% agar. The plates were incubated for two weeks to allow colony formation. Lastly, colonies were quantified using ImageJ, which provided a reliable and objective measurement of colony growth.

### Wound-healing assay

OSCC cells were seeded in 6-well culture plates and allowed to grow until reaching 90% confluence as a monolayer. Then, a sterile 200-μl pipette tip was gently and slowly stroked across the monolayer to create a linear scratch in each well's center. The plates were carefully washed with phosphate-buffered saline (PBS) solution to remove detached cells and debris. Next, the cells were treated with various imatinib concentrations for 24h, and the scratch was observed and documented using a microscope at regular intervals (0 and 9h). Microscopic images were captured to monitor cell migration into the scratched area over time.

### Trans-well assay

The cell invasion assay incorporated 24-well trans-well chambers with an 8.0-μm pore polycarbonate membrane insert (Corning Costar, Lowell, MA, USA). The insert's upper chamber was coated with BD Matrigel Matrix (BD Biosciences, NY, USA) to mimic the extracellular matrix. OSCC cells were seeded at 2 × 10^4^ cells per well in 100μl of serum-free media onto the Matrigel-coated membrane's upper surface, and the lower chamber was filled with media containing imatinib and 10% FBS as the chemoattractant for cell invasion. After incubation at 37 °C for 24h, non-invading cells on the membrane's upper surface were carefully removed, whereas the invaded cells on the lower surface were fixed with cold 4% formaldehyde, permeabilized with 100% methanol, and stained with 0.1% crystal violet. Membranes with invading cells were visualized using a Leica SP2 confocal microscope (Leica Microsystems, Wetzlar, Germany), and invaded cells were quantified with Image-Pro Plus.

### Cell apoptosis analysis

Imatinib-related apoptosis was assessed by seeding OSCC cells at a density of 1 × 10^4^ cells per well in 6-well culture dishes and allowing them to adhere overnight. The cells were treated with different imatinib concentrations over 48h and were then harvested and washed with 1 × cold PBS solution. The washed cells were centrifuged, and the supernatant was discarded. Next, the cells were resuspended in 1 × annexin-binding buffer and stained with annexin V (Invitrogen, Thermo Fisher Scientific, USA) and propidium iodide (PI) at room temperature for 15 min to visualize apoptotic cells. The stained cells were analyzed using the FACS Verse Flow Cytometry system (BD Science, CA, USA).

### Cell cycle analysis

For cell cycle analysis, OSCC cells were seeded at 1 × 10^5^ cells per well in 6-well culture dishes and incubated overnight in a 37 °C incubator for cell attachment and growth. Then, the cells were treated with different imatinib concentrations over 24h and were then harvested and washed twice with cold 1× PBS. To fix the cells, they were treated with 70% ethanol and incubated overnight at -20°C. Next, the cells were washed with cold 1 × PBS to remove residual fixative and stained with 0.5 mL of the FxCycleTM PI/RNase Staining Solution (Thermo Fisher Scientific) to label DNA content and enable cell cycle analysis. The staining solution was added to each flow cytometry sample and incubated at room temperature for 15-30 min, protected from light. Then, the samples were analyzed using a FACS Verse Flow Cytometry System (BD Sciences, CA, USA).

### Western blot assay

OSCC cells were seeded at a density of 1 × 10^6^ cells per 100-mm dish and treated with varying imatinib concentrations (0, 5, 10, and 20μM) for 24 h at 37°C. Following treatment, the cells were collected through centrifugation at 1000 rpm for 5min at 4°C and washed twice with PBS. Next, the cells were lysed using RIPA II cell lysis buffer (1×) supplemented with Triton without ethylenediaminetetraacetic acid (EDTA) and a protease inhibitor cocktail (100×). Cell lysate protein concentrations were determined with a BCA protein assay kit, and sodium dodecyl-sulfate polyacrylamide gel electrophoresis gels separated the protein samples (30μg). The separated proteins were transferred onto polyvinylidene fluoride membranes with a 0.22μm pore size. The membranes were blocked in 5% skim milk for 1h and incubated overnight at 4°C with target-specific primary antibodies. After incubation, the membranes were further incubated with the appropriate secondary antibodies for 1 h at room temperature. Immunoblots were visualized using the Thermo Scientific SuperSignal™ West Pico PLUS chemiluminescent substrate and imaged with the ImageQuant LAS 500 System.

### Tissue array

Fifth Generation Tissue Array (T-BO-1-TARP) Purchased from the National Cancer Institute Array program. Oral squamous cell carcinoma tissue chip containing marginal or paracancerous tissue. Each oral cavity squamous cell carcinoma tissue microarray containing 50 cases squamous cell carcinoma and 10 cases adjacent normal or cancer adjacent tissue, single core per case. The detailed parameter list is shown in Supplementary Table 1.

### Immunohistochemistry analysis

The sections used for immunohistochemistry (IHC) testing were paraffin-embedded sections (4μm). The slices of the tissue samples were then blocked with 1% BSA and treated with the primary antibodies PI3K (AF6242, Affinity Biosciences, Jiangsu, China) and p-PI3k (AF3242, Affinity Biosciences, Jiangsu, China) at 4 °C overnight. The tissue samples were deparaffinized, hydrated, and permeabilized with 0.5% Triton X-100 in 1 PBS for 10 mins. Cells were then given three PBS washes before being exposed to the proper secondary antibody. As directed by the manufacturer, 3, 3'-Diaminobenzidine (DAB) staining was utilized to visualize the protein targets. After 2 minutes of hematoxylin counterstaining, the cells were photographed under a microscope, analyzed, and quantified using Media Cybernetics' Image-Pro Plus software (v.6.1).

### Statistical analysis

All data were expressed as mean ± standard deviation (SD) and analyzed with SPSS (Version 25.0, IBM, Armonk, NY, USA). Student's t-test of variance was used to compare group values. All experiments were repeated thrice, and *P*-values < 0.05 were considered statistically significant.

## Results

### Imatinib inhibited OSCC cell growth

**Figure 1:**
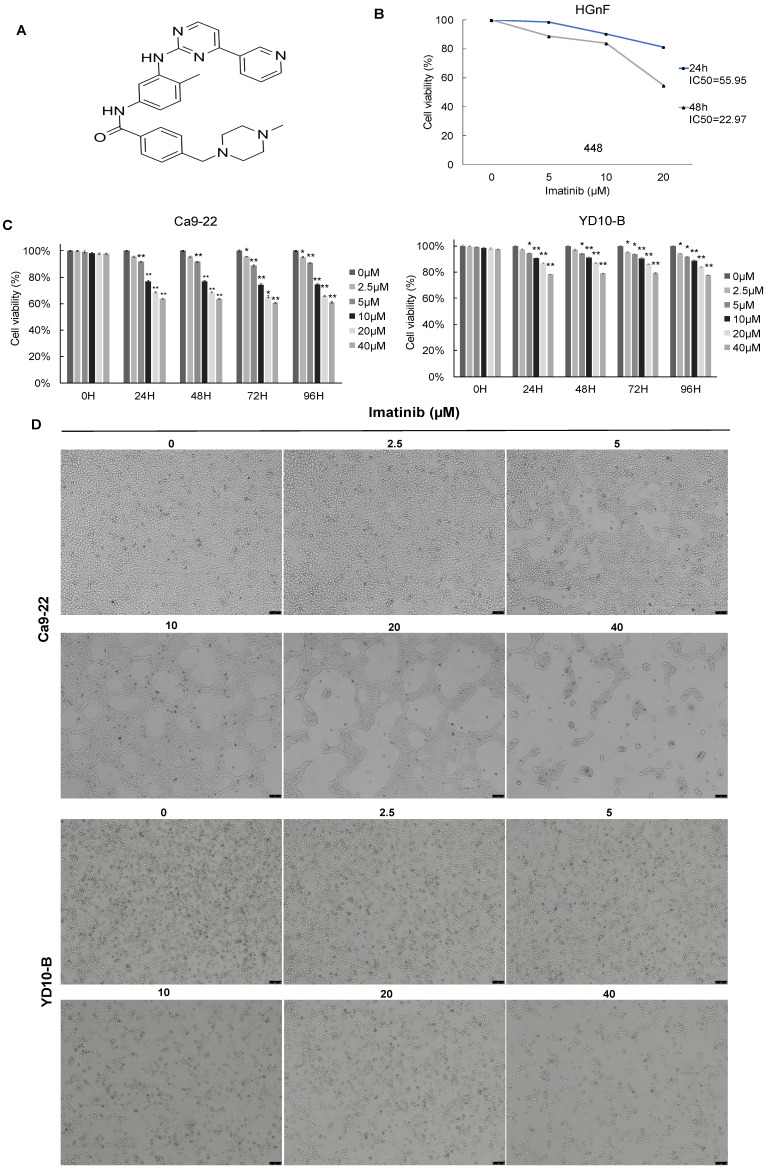

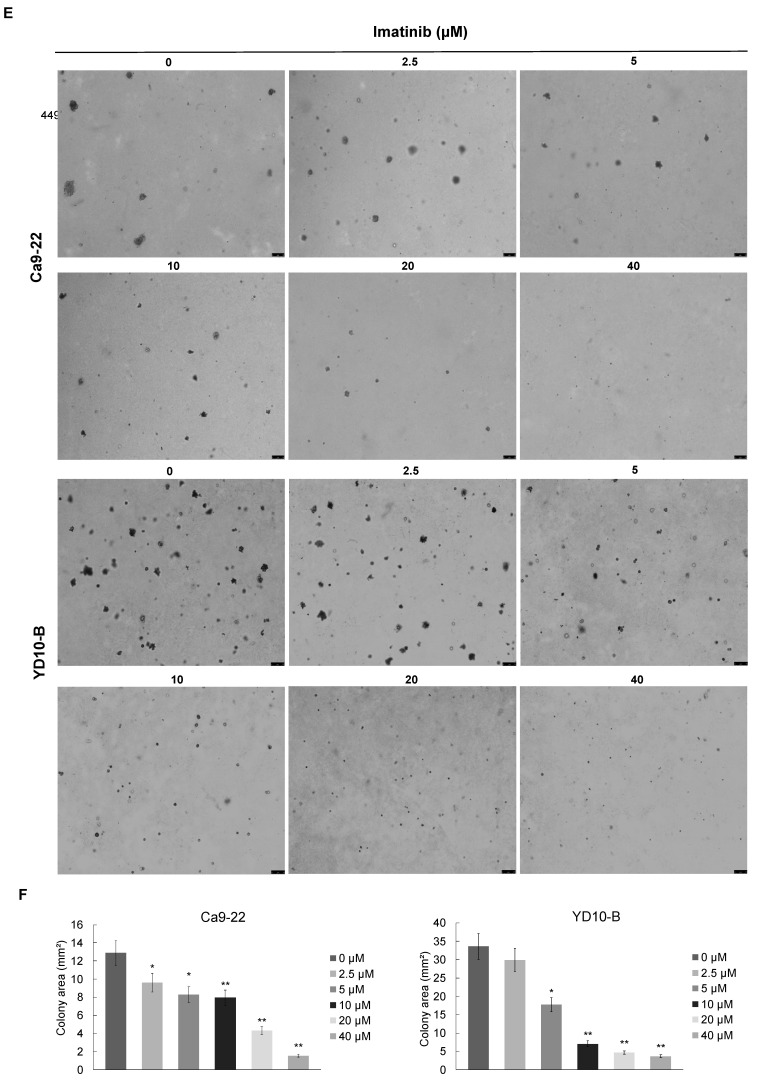
** Imatinib inhibits OSCC cell growth. (A)** Imatinib chemical structures. **(B)** Normal cell human gingival fibroblasts (HGnF) IC50 value was examined through CCK-8 assay for 24 h and 48h. **(C)** Cell viability analyzed with the CCK-8 assay. **(D)** Ca9-22 and YD-10B cell morphologies after 24h imatinib treatment, observed under a light microscope (magnification, 100×). **(E)** Anchorage-independent OSCC cell growth following imatinib treatment. **(F)** Quantitative graphs of colonies formed on soft agar by imatinib-treated YD-10B and Ca9-22 cells. Colonies were imaged using a microscope and quantified with Image-Pro PLUS (v.6). Data are shown as means ± standard deviation of values from three independent experiments, each with triplicate samples. The asterisk (*) indicates a significant (*P* < 0.05) and (**) represents a significant (*P* < 0.01) imatinib inhibitory effect.

The effect of imatinib (structure as shown in Figure 1A) on OSCC cell growth was investigated using different assays. The CCK-8 assay was used to test cell viability, and cell morphology analysis and colony formation assay were also completed. the IC50 of imatinib in HGnF over 24h and 48h were approximately 55.95 and 22.97μM, respectively. Therefore, 20μM of imatinib treated for 24h was selected to ensure no cytotoxic effect on the normal cells (Figure 1B). The CCK-8 assay results indicated that imatinib dose-dependently suppressed the Ca9-22 and YD-10B cell viability (Figure 1C), and the cell morphology analysis revealed that imatinib has an antiproliferative effect on OSCC cells. In addition, we observed cell morphology changes as imatinib-treated cells were more condensed and rounded than control cells. These findings indicate that imatinib may alter the cytoskeletal architecture of OSCC cells (Figure 1D). The colony formation assay was then conducted over two weeks with various imatinib concentrations for anchorage-independent cell growth assessment. Similarly, imatinib dose-dependently decreased colony number and size in both OSCC cell lines. The colony formation assay further confirmed the inhibitory effect of imatinib on OSCC cell growth (Figures 1E and 1F). These findings substantiate that imatinib does inhibit OSCC cell growth.

### Imatinib suppresses OSCC cell migration and invasion

**Figure 2:**
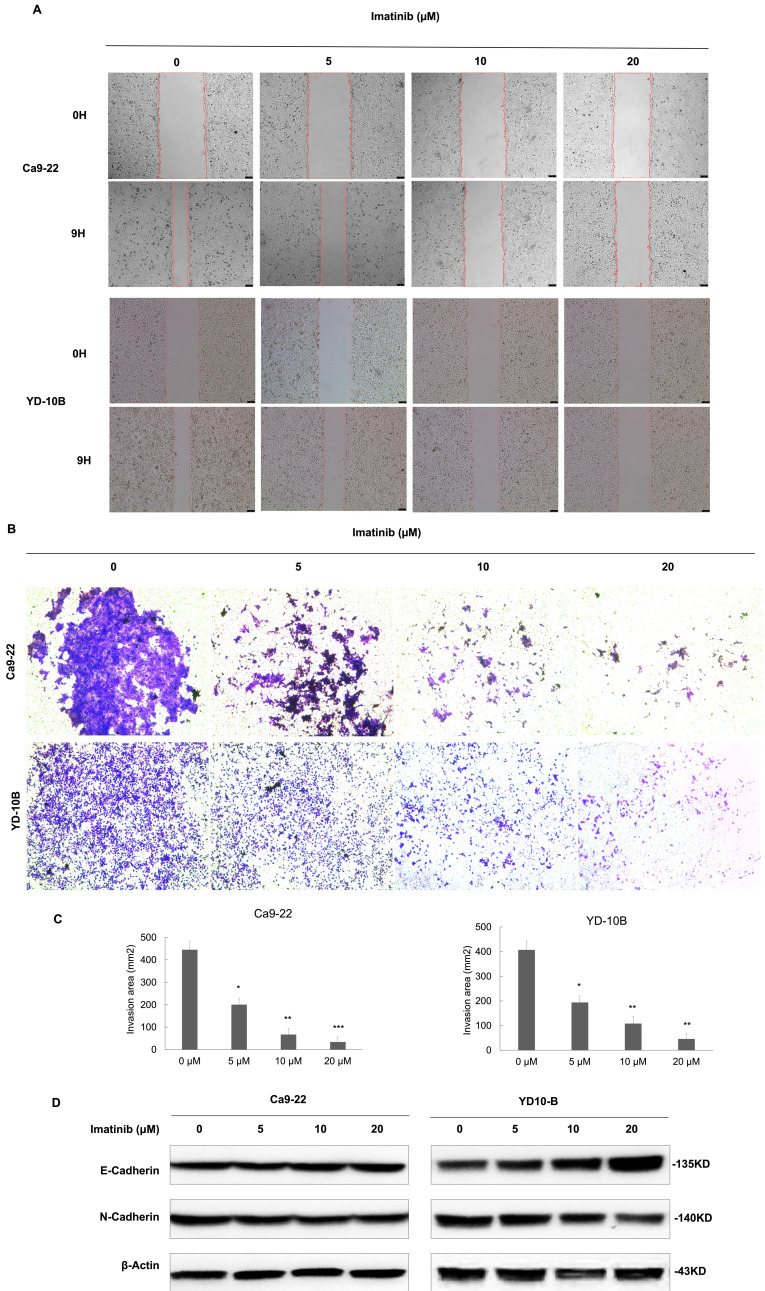
** Imatinib suppresses OSCC cell migration and invasion. (A)** Ca9-22 and YD-10B cells treated with imatinib or the DMSO control 0 and 9 h after wounding. **(B)** Representative images of invaded Ca9-22 and YD-10B cells treated with imatinib or the DMSO control. **(C)** Quantification of invaded cells for each treatment condition. **(D)** Representative Western blot images of E-cadherin and N-cadherin expression levels in Ca9-22 and YD- 10B cells treated with imatinib or the DMSO control. Β-actin was used as a loading control. Scale bar = 75μm. Data are shown as means ± standard deviation of values from three independent experiments, each with triplicate samples. *p < 0.05, **p < 0.01, and ***p < 0.001 compared with 0μM.

To clarify the effects of imatinib on OSCC metastasis, the migration and invasive abilities of OSCC cells were investigated. Four imatinib concentrations (0, 5, 10, and 20μM) were selected for experimentation based on the initial assays. The results verified that imatinib dose-dependently reduced migration compared with the control group (Figure 2A). Then, OSCC cells were treated with imatinib, and the trans-well assay was performed to evaluate the effect of imatinib on cell invasion. Similarly, these results substantiated that imatinib significantly inhibits OSCC cell invasion (Figure 2B and 2C). In addition, the Western blot analysis was completed to examine the epithelial-mesenchymal transition (EMT)-related markers E-cadherin and N-cadherin. Imatinib treatment upregulated E-cadherin and downregulated N-cadherin expressions, signifying that imatinib inhibited EMT in OSCC cells (Figure 2D). These results confirm the potential of imatinib as a promising OSCC treatment as it suppresses cell proliferation, migration, and invasion, which are vital for cancer progression.

### Imatinib induces OSCC cell apoptosis

**Figure 3:**
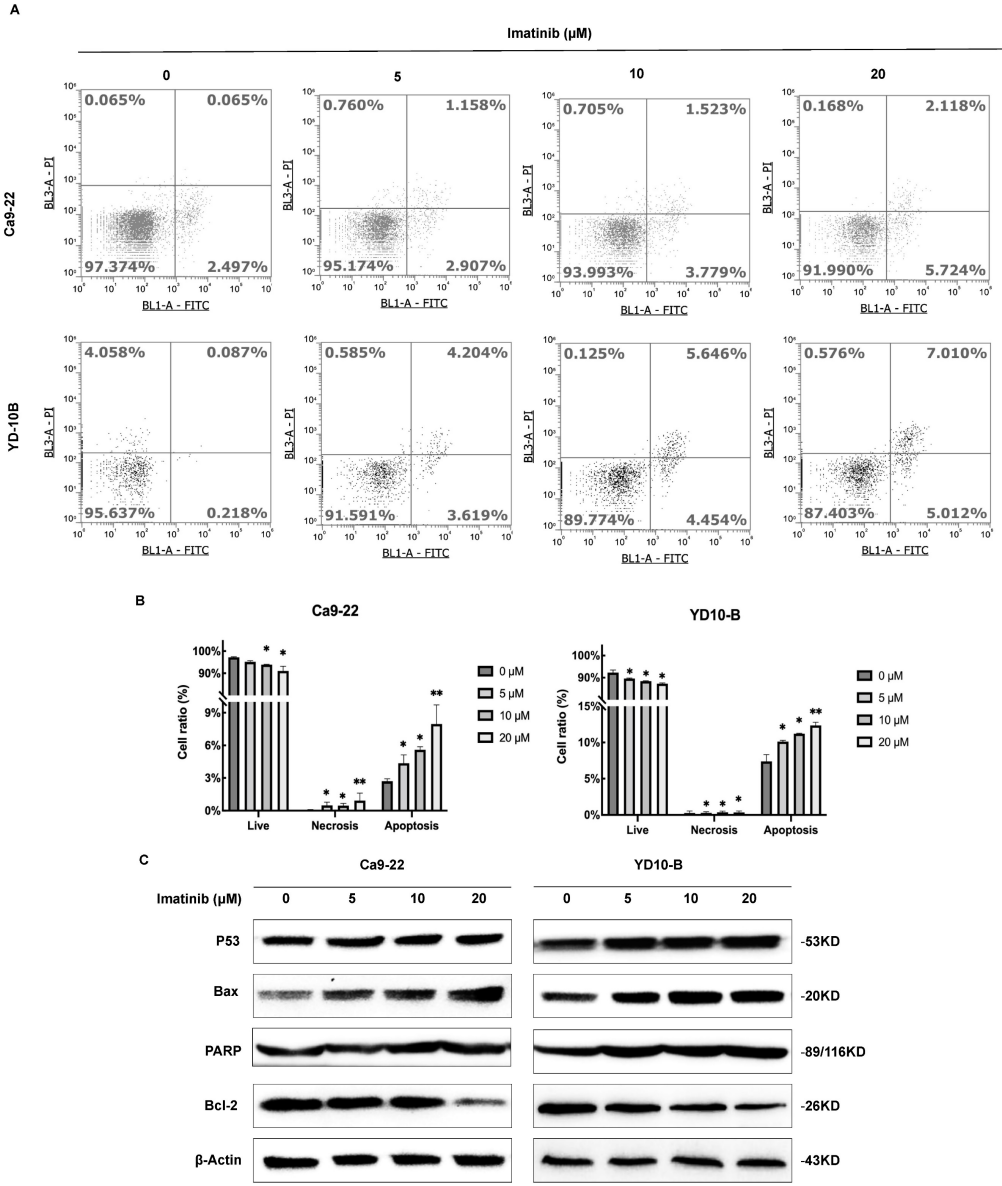
** Imatinib induces OSCC cell apoptosis. (A)** Representative flow cytometry images of Annexin V-FITC and PI staining in Ca9-22 and YD-10B cells treated with 0, 5, 10, and 20μM imatinib for 24 h. **(B)** Percentage of apoptotic cells in Ca9-22 and YD-10B cells treated with imatinib, as determined through flow cytometry analysis. **(C)** Representative Western blot images of p53, Bax, PARP, and Bcl-2 protein expression in Ca9-22 and YD-10B cells treated with imatinib. Data are shown as means ± standard deviation of values from three independent experiments, each with triplicate samples. *p < 0.05, **p < 0.01, and ***p < 0.001 compared with 0μM.

Imatinib-induced OSCC apoptosis was further investigated by analyzing apoptosis-related protein expression levels through flow cytometry and Western blotting. The flow cytometry analysis revealed that apoptotic cell percentage dose-dependently increased following imatinib therapy (Figures 3A and 3B). Western blotting analysis showed that p53, Bax, and PARP expression levels, which promote apoptosis, were also significantly upregulated dose-dependently (Figure 3C). Furthermore, Bcl-2 expression levels, an antiapoptotic protein, decreased following imatinib treatment. These findings collectively indicate imatinib-induced OSCC cell apoptosis by regulating pro- and antiapoptotic proteins (Figure 3C). Our results suggest that imatinib-induced apoptosis in OSCC cells is associated with apoptosis-related protein regulation, which activates the caspase-dependent apoptotic pathway.

### Imatinib induces OSCC cell apoptosis by suppressing the PI3K/AKT/mTOR signaling pathway

**Figure 4:**
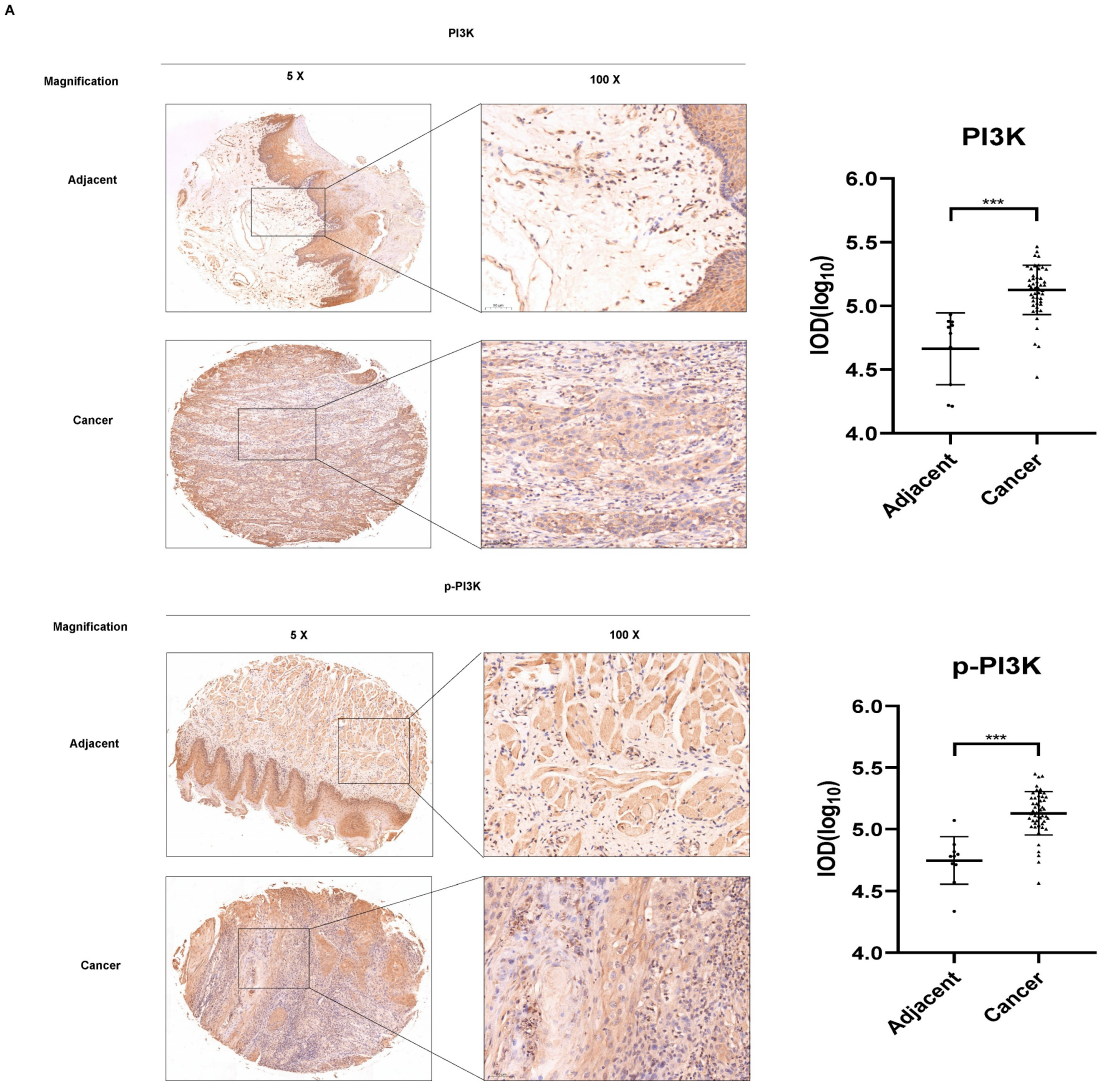

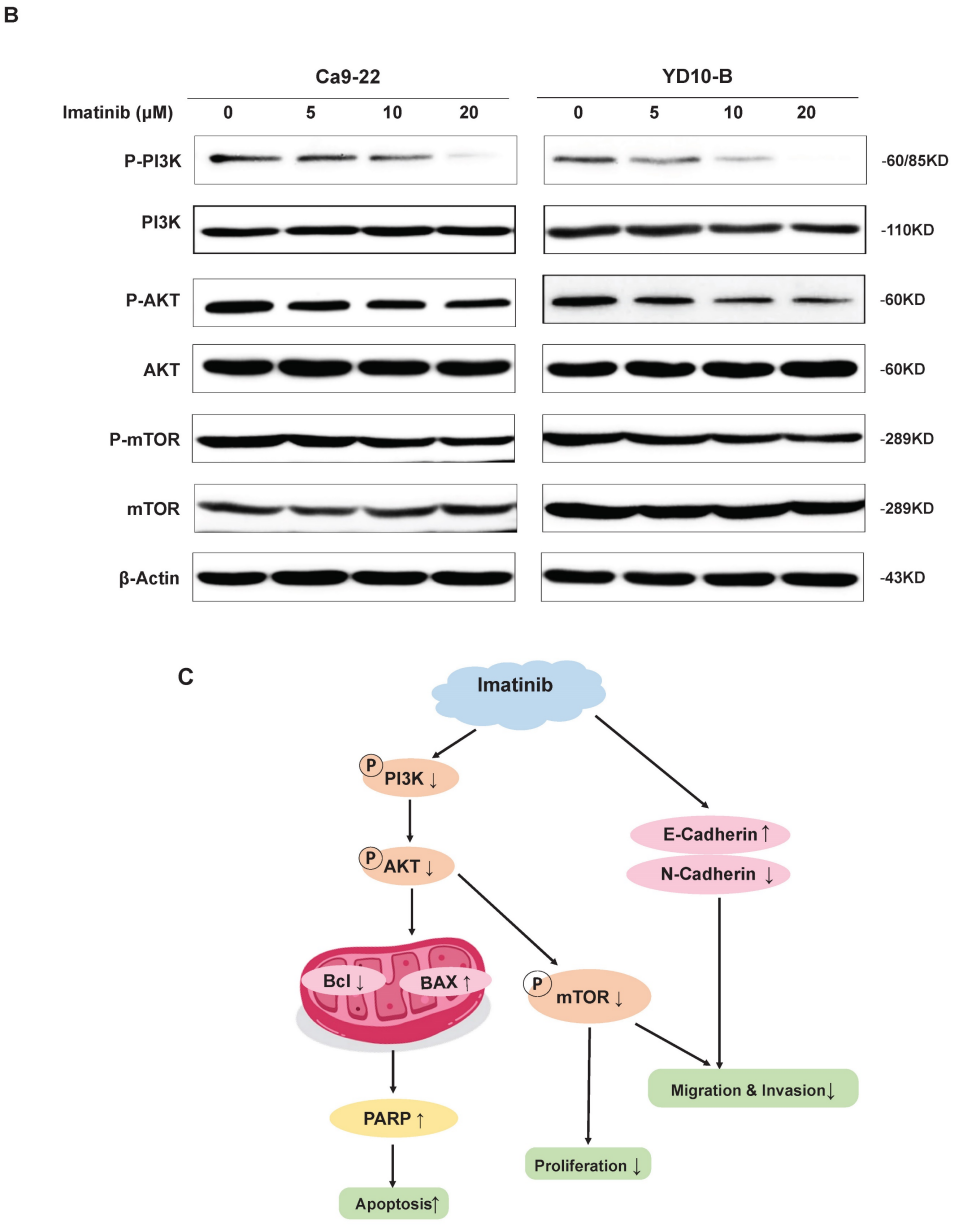
** Imatinib induces OSCC cell apoptosis via the PI3K/AKT/mTOR signaling pathway. (A)** The expressions of PI3K and p-PI3K were examined by IHC analysis (100× magnification).** (B)** PI3K, p-PI3K AKT, p-AKT, mTOR, and p-mTOR expression levels were determined through Western blotting. **(C)** Imatinib induces OSCC cell apoptosis and suppression via the PI3K/AKT/mTOR signaling pathway.

As evidenced by the elevated p53, Bax, and PARP expression levels (Figure 3C), our results demonstrated that imatinib effectively induced OSCC cell apoptosis. Therefore, we aimed to elucidate the mechanism underlying these results. Phosphatidylinositol 3-kinase (PI3K)/Akt/mammalian target of rapamycin (mTOR) signaling pathway is one of the major cellular signaling pathways that plays an important role in basic intracellular functions. Irregularities in the major components of the PI3K/AKT/mTOR signaling pathway are common in human cancers. To evaluate the role of PI3K in OSCC, we examined the expression of total and phosphorylated PI3K in an OSCC tumor microarray. We found that in OSCC cancer tissues, PI3K and p-PI3K expressions increased significantly compared with adjacent normal or adjacent to cancer tissues (Figure 4A). These results indicated PI3K signal pathway may plays important role in OSCC cancer. We wonder that whether imatinib inhibited OSCC cell growth by inhibiting PI3K signaling pathway. Western blot analysis was utilized to examine expression levels of critical proteins involved in the PI3K/AKT/mTOR signaling pathway, including PI3K, p-PI3K, AKT, p-AKT, mTOR, and p-mTOR. Notably, phosphorylated PI3K, AKT, and mTOR forms were downregulated in imatinib-treated cells compared with the control group (Figure 4B). This finding proves that imatinib exerts apoptotic effects on OSCC cells by suppressing the PI3K/AKT/mTOR signaling pathway (Figure 4C). Thus, inhibiting this pathway could impede cell survival and growth, engendering apoptosis.

## Discussion

OSCC is a prevalent cancer with aggressive behavior and limited treatment options 13. Surgery, radiation, chemotherapy, and other current therapeutics have limited efficacy, and overall advanced OSCC survival rates remain poor 14. Therefore, exploring novel OSCC therapeutic strategies is crucial. Imatinib, a known anti-cancer agent, has displayed promising clinical efficacy against various cancer types 15. Previous studies have demonstrated imatinib's therapeutic effects on assorted cancers, such as leukemia and gastrointestinal stromal tumors 16, 17. However, its specific impact on OSCC has yet to be fully elucidated. Therefore, this study investigated the anticancer effects of imatinib on Ca9-22 and YD10-B OSCC cell lines.

OSCC cell proliferation fundamentally drives tumor growth and progression. Previous studies have demonstrated the antiproliferative effects of imatinib on various cancer types, such as leukemia and gastrointestinal stromal tumors 18, 19, and we discovered that imatinib also effectively inhibits OSCC cell proliferation, as evidenced by the dose- and time-dependent reductions in cell viability (Figures 2A and 2B). These findings establish that the inhibitory effect of imatinib on cell proliferation is a consistent effect among cancers, including OSCC. However, the specific molecular mechanisms underlying this inhibition may vary by cancer type. Nonetheless, the ability of imatinib to inhibit OSCC cell proliferation highlights its potential as a therapeutic agent for controlling tumor growth in patients with OSCC.

EMT is integral for promoting cancer cell invasion and metastasis 20. OSCC enhances migratory and invasive properties, augmenting its metastatic potential 21. This study investigated the effect of imatinib on OSCC cell migration and invasion, revealing that imatinib suppressed OSCC cell invasion and migration (Figures 2A and 2B). In addition, imatinib upregulated E-cadherin and downregulated N-cadherin expressions, indicating that imatinib inhibited EMT in OSCC cells (Figure 2C). These results propose that imatinib interferes with the EMT process, providing a potential mechanism for its OSCC anti-invasion and anti-migration effects.

Apoptosis is a tightly regulated programmed cell death that is dysregulated in cancer cells, prompting uncontrolled cell survival and proliferation 22. Therefore, we also verified that imatinib induced OSCC cell apoptosis through a flow cytometry analysis (Figures 3A and 3B). Moreover, apoptosis-related protein expression levels were detected to understand this underlying mechanism. Our results showed that imatinib increased proapoptotic protein expression levels, including p53 and Bax, while decreasing Bcl-2 expression, an antiapoptotic protein (Figure 3C). These results corroborate previous studies that have identified the proapoptotic effects of imatinib on various cancers 23. These findings support that imatinib promotes OSCC cell apoptosis by modulating the balance between pro- and antiapoptotic proteins.

The PI3K/AKT/mTOR signaling pathway is critical in regulating cell survival, proliferation, and growth in various cellular contexts, including cancer. This pathway is frequently dysregulated in cancer, effectuating aberrant activation and promoting tumor development and progression 24. PI3K activation phosphorylates AKT, which in turn phosphorylates and activates mTOR. Then, the activated mTOR regulates downstream effectors involved in protein synthesis, cell cycle progression, and survival 25. Thus, this study investigated the effect of imatinib on the PI3K/AKT/mTOR signaling pathway in OSCC cells. These results demonstrated that imatinib inhibited PI3K, AKT, and mTOR phosphorylation, indicating this suppression effect on signaling pathway (Figure 4A), corroborating previous studies that reported the inhibition effect of imatinib on the PI3K/AKT/mTOR pathway in various cancers 26. Imatinib-related suppression of the PI3K/AKT/mTOR signaling pathway has significant implications for treating OSCC, as the dysregulated activation of this pathway contributes to cell survival, proliferation, and resistance to apoptosis. Thus, by targeting this signaling pathway, imatinib may disrupt the pivotal signaling cascades involved in OSCC progression, reducing cell survival and promoting apoptosis. Furthermore, imatinib-related modulation of the PI3K/AKT/mTOR pathway may have implications beyond OSCC. Previous studies have demonstrated this pathway's involvement in various cancers, highlighting its significance as a therapeutic target 26. The ability of imatinib to suppress this pathway verifies its potential as a multitargeted agent for cancer treatment.

This study revealed that imatinib inhibits OSCC cell proliferation, suppresses migration and invasion, and induces apoptosis by modulating the PI3K/AKT/mTOR signaling pathway. This inhibition likely contributes to observable effects of imatinib on cell survival, proliferation, and apoptosis in OSCC. Therefore, targeting this pathway provides a promising strategy for OSCC therapy.

## Supplementary Material

Supplementary figures and table.
